# Genetic Modifiers of Duchenne Muscular Dystrophy and Dilated Cardiomyopathy

**DOI:** 10.1371/journal.pone.0141240

**Published:** 2015-10-29

**Authors:** Andrea Barp, Luca Bello, Luisa Politano, Paola Melacini, Chiara Calore, Angela Polo, Sara Vianello, Gianni Sorarù, Claudio Semplicini, Boris Pantic, Antonella Taglia, Ester Picillo, Francesca Magri, Ksenija Gorni, Sonia Messina, Gian Luca Vita, Giuseppe Vita, Giacomo P. Comi, Mario Ermani, Vincenzo Calvo, Corrado Angelini, Eric P. Hoffman, Elena Pegoraro

**Affiliations:** 1 Neuromuscular Center, Department of Neuroscience, University of Padova, Padova, Italy; 2 Department of Experimental Medicine, Cardiomyology and Medical Genetics, Second University of Naples, Naples, Italy; 3 Department of Cardiac, Thoracic and Vascular Sciences, Cardiology Section, University of Padova, Padova, Italy; 4 Dino Ferrari Centre, Department of Neurological Sciences, University of Milan, I.R.C.C.S. Foundation Cà Granda, Ospedale Maggiore Policlinico, Milan, Italy; 5 NEuroMuscular Omnicentre (NEMO), Fondazione Serena Onlus, Ospedale Niguarda Cà Granda, Milano, Italy; 6 Department of Neurosciences, Psychiatry and Anaesthesiology, University of Messina, Messina, Italy; 7 Department of Philosophy, Sociology, Pedagogy and Applied Psychology (FISPPA), University of Padova, Padova, Italy; 8 Istituto di Ricovero e Cura a Carattere Scientifico (IRCCS) San Camillo, Venice, Italy; 9 Research Center for Genetic Medicine, Children’s National Medical Center, 111 Michigan Avenue, NW, Washington, DC, 20010, United States of America; University of Louisville School of Medicine, UNITED STATES

## Abstract

**Objective:**

Dilated cardiomyopathy (DCM) is a major complication and leading cause of death in Duchenne muscular dystrophy (DMD). DCM onset is variable, suggesting modifier effects of genetic or environmental factors. We aimed to determine if polymorphisms previously associated with age at loss of independent ambulation (LoA) in DMD (rs28357094 in the *SPP1* promoter, rs10880 and the VTTT/IAAM haplotype in *LTBP4*) also modify DCM onset.

**Methods:**

A multicentric cohort of 178 DMD patients was genotyped by TaqMan assays. We performed a time-to-event analysis of DCM onset, with age as time variable, and finding of left ventricular ejection fraction < 50% and/or end diastolic volume > 70 mL/m^2^ as event (confirmed by a previous normal exam < 12 months prior); DCM-free patients were censored at the age of last echocardiographic follow-up.

**Results:**

Patients were followed up to an average age of 15.9 ± 6.7 years. Seventy-one/178 patients developed DCM, and median age at onset was 20.0 years. Glucocorticoid corticosteroid treatment (n = 88 untreated; n = 75 treated; n = 15 unknown) did not have a significant independent effect on DCM onset. Cardiological medications were not administered before DCM onset in this population. We observed trends towards a protective effect of the dominant G allele at *SPP1* rs28357094 and recessive T allele at *LTBP4* rs10880, which was statistically significant in steroid-treated patients for *LTBP4* rs10880 (< 50% T/T patients developing DCM during follow-up [n = 13]; median DCM onset 17.6 years for C/C-C/T, log-rank p = 0.027).

**Conclusions:**

We report a putative protective effect of DMD genetic modifiers on the development of cardiac complications, that might aid in risk stratification if confirmed in independent cohorts.

## Introduction

Duchenne muscular dystrophy (DMD) is a lethal, progressive neuromuscular disease due to *DMD* gene mutations resulting in a complete lack of dystrophin in the skeletal muscle and myocardium[1]. Dilated cardiomyopathy (DCM) is a significant clinical feature of DMD, and increasing utilization of nocturnal ventilation has led to a greater proportion of DMD patients succumbing to DCM-related cardiac failure, in parallel to reduced mortality due to respiratory insufficiency[2]. DCM onset is variable: minor electrocardiographic alterations are usually detectable from the age of 10, evolving towards DCM with biventricular dilation and depression of left ventricular ejection fraction. By the end of the second decade, most patients exhibit cardiac insufficiency[3]. DCM progression is also variable, with no obvious correlation to muscle weakness. Indeed, some authors argue that weaker patients show better preservation of myocardial function, due to less demand on the heart[3,4].

Glucocorticoid corticosteroids (hereafter “steroids”) are the only available pharmacological therapy able to slow the progression of muscle weakness in DMD[5], but there are contradictory reports on their effect on cardiac function. A protective effect in slowing DCM onset and progression has been reported by some authors[6–8], but denied by others[9,10]. Furthermore, steroids damage the myocardium in animal models of muscular dystrophy, exacerbating fibrosis[11–13].

Osteopontin (OPN), encoded by the *Secreted PhosphoProtein 1* (*SPP1*) gene, is a cytokine involved in inflammation and tissue remodeling[14]. OPN is expressed by different cell types, including myoblasts, in the *mdx* mouse muscle[15] and regulates inflammatory infiltration and muscle regeneration[16]. Moreover, *SPP1* genetic ablation in the *mdx* mouse induces a milder disease course and a decrease in myocardial and diaphragmatic fibrosis through a reduction of TGFβ (Transforming Growth Factor β)[17], which is itself a strong activator of the *SPP1* promoter[18]. OPN is upregulated in dystrophic muscle[16,19–21], and, interestingly, is also a biomarker and mediator of cardiovascular disease[22]. Its overexpression in the murine myocardium causes myocarditis and DCM[23]. The G allele at the single nucleotide polymorphism (SNP) rs28357094, in the *SPP1* promoter, was associated with more severe weakness in three independent DMD cohorts[19,24,25], although other authors failed to confirm this[26,27]. Underlying molecular mechanisms involve transcriptional regulation[28] and interactions with other pro-inflammatory factors, such as TGFβ[21]. Recent studies have shown the SNP to be steroid dependent, both *in vitro*[29] and *in vivo*[25], suggesting a pharmacogenetic mechanism.

The latent TGFβ binding protein 4 (*Ltbp4*) locus showed linkage with disease severity in a mouse model of muscular dystrophy[30], and a common haplotype in the human *LTBP4* gene, encoding different isoproteins, was found to modify age at loss of ambulation (LoA) in a cohort of patients with severe dystrophinopathy[26]. This finding was validated in independent cohorts[25,27]. As LTBP4 binds TGFβ in a latent complex in the extracellular matrix, preventing it from reaching its cell surface receptors, the proposed mechanism is that the protective haplotype renders the complex more stable, preventing pro-fibrotic TGFβ signaling[31,32].

Here we test the hypothesis that *SPP1* and *LTBP4* modify DCM onset in DMD.

## Materials and Methods

### Inclusion criteria

Inclusion criteria were as following: a) confirmed diagnosis of DMD (out-of-frame/nonsense *DMD* gene mutations and/or absence of dystrophin by immunohistochemistry or western blot of muscle tissue); b) records of a regular (annual) cardiologic follow-up, including 2D-M-mode echocardiography; b) availability of a DNA sample.

### Informed consent and ethics

All the patients or their guardians gave written informed consent to use of DNA samples and medical record data (including results of echocardiograms) for research purposes, at all participating institutions which provided DNA samples (Universities of Padova, Naples, Messina and Milan; NEuroMuscular Omnicenter, Milan). The study was approved by the Ethics Committee at each institution where patients were recruited (Comitato Etico per la Sperimentazione dell'Azienda Ospedaliera di Padova, Comitato Etico dell'Azienda Ospedaliera Universitaria della Seconda Università di Napoli, Comitato Etico Interaziendale della Provincia di Messina, Comitato di Etica e Sperimentazione Farmacologica IRCCS Ca' Granda Ospedale Maggiore Policlinico, Comitato Etico Milano Area C), in compliance with the Declaration of Helsinki.

### Steroid treatment and cardiological treatments

Patients were categorized as “steroid treated” if treated for at least one year with a standard dose of oral prednisone or equivalent dose of deflazacort (0.75 mg/kg/day; 0.9 mg/kg/day respectively) before events (DCM onset or LoA) or censoring. In this population, patients were not treated before DCM onset with prophylaptic cardiological medications such as angiotensin converting anzyme inhibitors (ACEi), angiotensin receptor blockers (ARB), or beta-blockers.

### Echocardiographic studies

Echocardiographic studies were performed with Philips SONOS 5500 instruments with a 3 MHz transducer or equivalent instruments. Two-dimensional images and M-mode echocardiograms of atrial and ventricular cavities were obtained in multiple cross-sectional planes, with the transducer in standard positions according to the recommendations of the American Society of Echocardiography[33,34]. Left ventricular (LV) ejection fraction (EF) was calculated from two-dimensional images with modified Simpson’s formula or area–length method[33].

### Definition of DCM onset

DCM onset was defined as the age at the first echocardiographic finding of LV end diastolic volume (EDV) > 70 mL/m^2^, and/or LV-EF < 50%. We excluded patients whose first abnormal echo was not preceded by a normal one < 12 months prior, because age at DCM onset could not be established with certainty in these cases. Patients with a normal ecocardiographic follow-up were considered “censored” at the age of the last echo.

### Genotyping and inheritance models

Genotypes at the SNPs rs28357094 (T/G nucleotide substitution at position -66 in the promoter region of the *SPP1* gene), rs2303729 (LTBP4 V194I), rs1131620 (LTBP4 T787A), and rs10880 (LTBP4 T1140M) were determined by Applied Biosystems TaqMan SNP genotyping assays and end-point allelic discrimination on an ABI-7000 SDS instrument. In the determination of LTBP4 haplotypes, genotype at the fourth SNP rs1051303 (T820A) was imputed from rs1131620 genotype, assuming no recombination events due to very strong linkage disequilibrium (LD). Haplotypes were phased by PLINK[35]. Patients were assigned to genotype groups according to previously described inheritance models: dominant for rs28357094[19], and recessive for rs10880[26]. Additionally, patients were grouped based on LTBP4 haplotype: homozygotes for the VTTT haplotype (rs2303729 G, rs1131620 A, rs10880 C), homozygotes for the IAAM haplotype (rs2303729 A, rs1131620 G, rs10880 T), or other.

### Statistical analyses

The relation between age at LoA, age of DCM onset, steroid treatment and genotype at rs28357094, rs2303729, rs1131620, rs10880, and *LTBP4* haplotype was studied.

We used the Kaplan-Meier nonparametric method to estimate the survivor distribution functions of age at LoA and DCM onset. The log-rank test was used to test the significance of effects of genotype and steroid therapy. Linear correlations between 2 variables was tested by Pearson r. Concurrent effects of genotypes and age on LV-EF and LV-EDV (cross-sectional analysis) were evaluated by analysis of variance (ANOVA) in multiple linear regression models with EF or EDV as dependent variables, and age + genotype (dominant model for rs28357094 and recessive model for rs10880) as predictors. For all analyses, 2-tailed p values of less than 0.05 were considered significant. Analyses were done with SPSS version 18.0, R version 3.2.1, and Partek Genomics Suite 6.6. Based on Caucasian allele frequencies, we estimate that in our population statistical power for detection of SNP or haplotype effect is 0.8 with a median genotype-related difference in age at onset of DCM of 10 years for *SPP1* rs28357094 (dominant model), and 12 years for *LTBP4* haplotype (recessive model).

## Results

### Patients

One hundred and seventy-eight patients selected according to inclusion criteria (see Methods) were followed up to an average age of 15.9±6.7 years. Seventy-five/178 (42.1%) were steroid-treated, while 88/178 (49.4%) were untreated (or treated <1 year). For 15 patients (8.5%) information about treatment was unavailable or insufficiently detailed (duration, dose adequacy, treatment before-after events etc).

### *SPP1* rs28357094 genotyping

There were 111 homozygotes for the T allele (62.4%), 59 T/G heterozygotes (33.1%) and 8 homozygotes for the G allele (4.5%). This distribution was close to expected minor allele frequency (MAF) in a Caucasian population (21.1%) and consistent with Hardy-Weinberg equilibrium (HWE).

### *LTBP4* genotyping

One hundred and sixty-eight/178 patients were genotyped for *LTBP4* SNPs. Genotyping results were as follows: rs2303729 68 G/G, 66 G/A, 32 A/A (MAF 39.2%, HWE p = 0.035), rs1131620 61 A/A, 67 A/G, 39 GG (MAF, 43.4%, HWE p = 0.018), and rs10880 68 CC, 69 C/T, 31 T/T (MAF 39.0%, HWE p = 0.07). Total patient numbers do not coincide exactly because of limited DNA availability in a few patients. LTBP4 haplotype could be phased for 166 patients. Haplotype frequencies were 50.1% VTTT, 27.3% IAAM, 8.8% IAAT, 6.7% VAAM, and 6.9% pooled rare haplotypes (including VTTM, ITTT and ITTM). Forty-nine patients (29.5%) were homozygotes for the VTTT haplotype, and 16 (9.6%) for the IAAM haplotype. These findings were close to the expected distribution for a Caucasian population.

### DCM natural history

**Fig 1:**
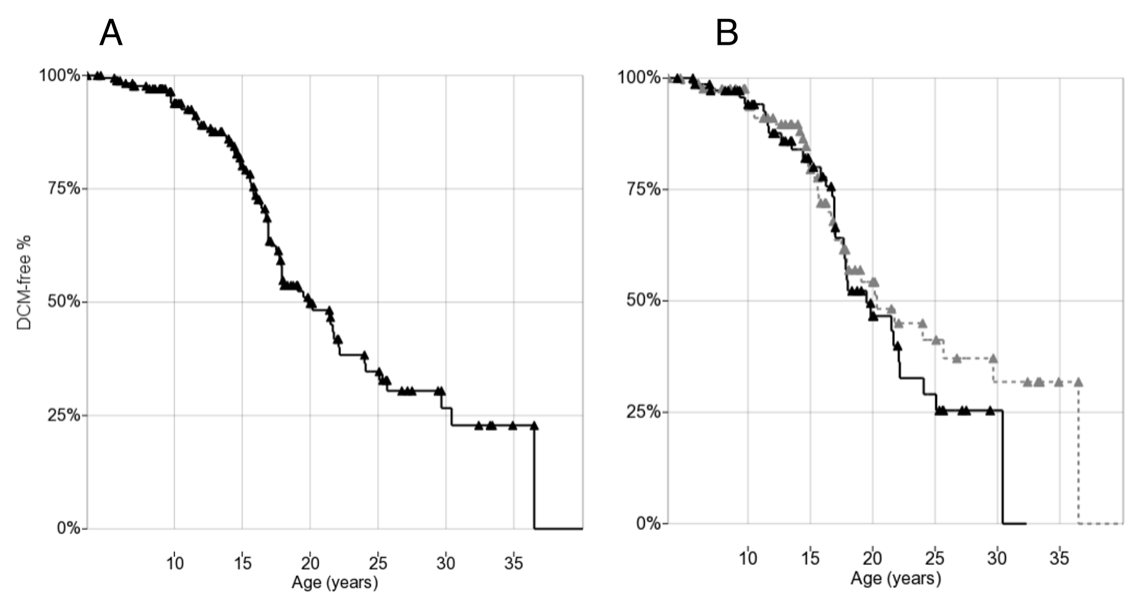
(A) Kaplan-Meier plot of DCM onset in 178 DMD patients. (B) Kaplan-Meier plot of DCM onset by steroid treatment: solid treated (>1 year before event/censoring), dashed untreated. Triangles indicate censoring.

Seventy-one/178 patients (40%) developed DCM (as defined by echocardiographic criteria) during follow-up. Of these, 32 had both LV-EF and EDV available, 28 EF only, and 11 EDV only. Of the 32 patients who had both LV-EF and EDV available in the first pathological echocardiogram, both measures were altered in 15/32 (47%), EDV only with normal EF in 9/32 (28%), and EF only with normal EDV in 8/32 (25%). Mean age at onset in patients who presented with DCM was 16.5±6.0 years (range 5.4–40.1 years), with no significant differences between EF-defined and EDV-defined. In the 107 patients (60%) who did not develop DCM during the observation period (“censored”), mean age at last normal echocardiography was 15.5±7.1 years (range 3.6–36.5 years). Kaplan-Meier plots for DCM showed a median age onset at 20.0 years (95% confidence interval: 17.4–22.6; first quartile 30.5 and third 16.0 years) (Fig 1A).

### Steroid therapy and DCM

Kaplan-Meier plots comparing DCM-free survival between steroid-treated (n = 75) and untreated patients (n = 88) did not show significant differences (median onset 20.0 years vs. 20.5 years respectively, Fig 1B).

### Genotypes and DCM

**Fig 2:**
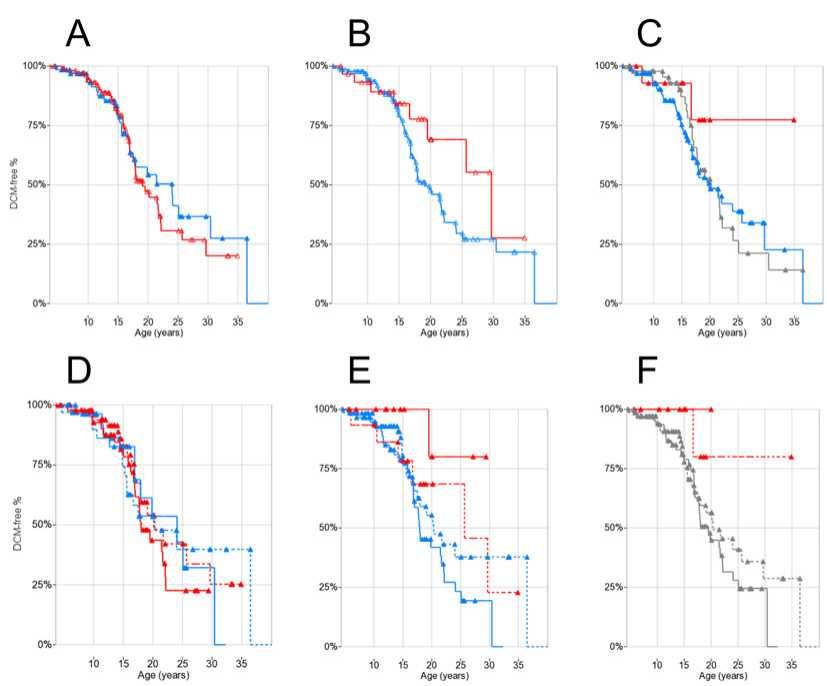
Kaplan-Meier plots of DCM onset by genotypes and steroid treatment. Triangles indicate censoring. (A) *SPP1* rs28357094: T/T red, T/G-G/G blue; (B) *LTBP4* rs10880: T/T red, C/C-C/T blue; (C) LTBP4 haplotype: IAAM/IAAM red, VTTT/VTTT grey, other blue; (D) *SPP1* rs28357094 and steroid treatment: T/T red, T/G-G/G blue, solid treated (>1 year before event/censoring), dashed untreated; (E) rs10880 and steroid treatment: T/T red, C/C-C/T blue, solid treated, dashed untreated; (F) LTBP4 haplotype and steroid treatment: IAAM/IAAM red, other (including VTTT) grey, solid treated, dashed untreated.

The cross-sectional analysis of LV-EF and LV-EDV values by age and genotype showed no significant correlations (ANOVA p = n.s. for both age and genotype; S1 and S2 Figs). Kaplan-Meier plots showed difference in estimated medians for DCM onset between rs28357094 genotypes: 24.1 years for T/G-G/G (n = 111) and 19.1 for T/T (n = 67), although not statistically significant (Fig 2A). There was a trend towards later onset of DCM in patients carrying the *LTBP4* rs10880 T/T vs. C/C-C/T genotype (median 29.5 vs.19.0 years, n = 31 and 137 respectively, log-rank p = 0.13) (Fig 2B). Findings for other *LTBP4* SNPs were similar due to LD, and also not significant (data not shown). The IAAM/IAAM haplotype showed a trend of association to later DCM onset (>50% DCM-free patients at last follow-up vs. 20.0 years at median onset, n = 16 and 150 respectively, log-rank p = 0.15) (Fig 2C).

### Steroid therapy and genotype concurrent effects

Kaplan-Meier plots with patient grouping by genotype x steroid treatment did not show significant differences between *SPP1* genotypes in treated-untreated patients (Fig 2D). On the other hand, when grouping by *LTBP4* rs10880 x steroid treatment, within the steroid-treated group there was a significant difference between C/C-C/T genotype (median DCM onset 17.9 years, n = 60) and T/T genotype (>50% of patients DCM-free at last follow-up, n = 13, log-rank p = 0.027) (Fig 2E). When grouping by *LTBP4* haplotype, no DCM onset events were observed in 6 IAAM/IAAM steroid-treated patients, while median onset of DCM in 67 steroid-treated patients carrying other haplotypes was 19.0 years; however this difference was not significant (log-rank p = 0.26) (Fig 2F). Results regarding DCM onset are summarized in Table 1.

**Table 1:** Median age at DCM onset by *SPP1* and *LTBP4* genotype.

		*SPP1* rs28357094		*LTBP4* rs10880	
	All patients	T/T	T/G-G/G	C/C-C/T	T/T
Median age at DCM onset	20.0 years (n = 178)	19.1 years (n = 111)	24.1 years (n = 67)	19.0 years (n = 137)	29.5 years (n = 31)
Median age at DCM onset in steroid treated	20.0 years (n = 75)	17.0 years (n = 45)	24.0 years (n = 30)	*17*.9 years (n = 60)*	***< 50% DCM*** ^**§**^ (n = 13)*
Median age at DCM onset in untreated	20.5 years (n = 88)	20.1 years (n = 54)	20.2 years (n = 34)	20.2 years (n = 65)	25.8 years (n = 15)

*Significant difference between genotypes (log-rank p<0.05).

§DCM onset was observed in less than 50% of patients, so no median value can be calculated.

Total n for *LTBP4* differs due to limited DNA availability in a few patients. For *SPP1*, patients included in the previous report about loss of ambulation (Pegoraro et al, 2011) were also excluded. *SPP1*: Secreted PhosphoProtein 1. *LTBP4*: latent transforming growth factor beta binding protein 4. DCM: dilated cardiomyopathy.

### Loss of ambulation

Age at LoA was available for 163/178 patients, of whom 145 did not have severe cognitive impairment or other medical conditions potentially affecting age at LoA (e.g. bone fractures, prolonged immobilization). These patients were selected for association analysis between genotypes and LoA (“LoA cohort”). For *SPP1*, we also excluded 22/145 patients who were previously included in the original report of association between *SPP1* rs28357094 genotype and LoA in DMD[19].

**Fig 3:**
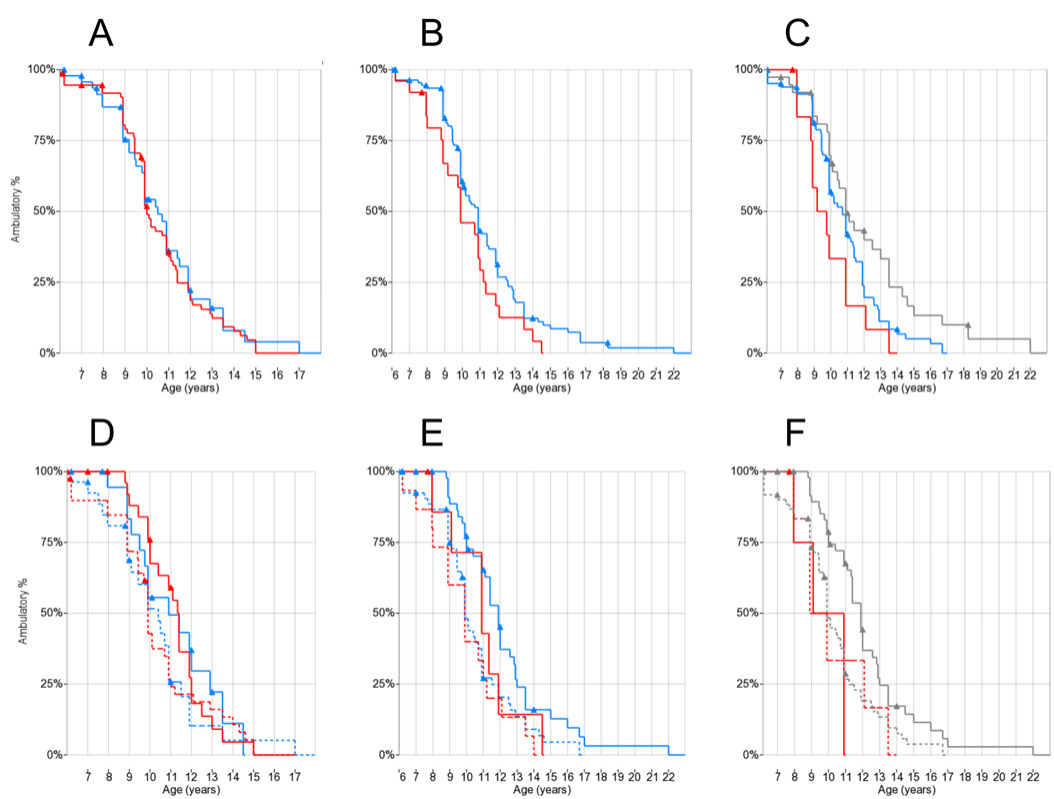
Kaplan-Meier plots of LoA by genotypes and steroid treatment. Triangles indicate censoring. (A) *SPP1* rs28357094 genotype: T/T red, T/G-G/G blue; (B) *LTBP4* rs10880: T/T red, C/C-C/T blue; (C) LTBP4 haplotype: IAAM/IAAM red, VTTT/VTTT grey, other blue; (D) rs28357094 genotype and steroid treatment: T/T red, T/G-G/G blue, solid treated (>1 year before LoA), dashed untreated; (E) rs10880 and steroid treatment: T/T red, C/C-C/T blue, solid treated, dashed untreated; (F) LTBP4 haplotype and steroid treatment: IAAM/IAAM red, other (including VTTT) grey, solid treated, dashed untreated.

Kaplan-Meier analysis showed no significant difference in median ages at LoA between *SPP1* rs28357094 genotypes (T/T 10.0 years, n = 81, T/G-G/G 10.5 years, n = 55) (Fig 3A). Of these 123 patients, 47 (38.2%) had been treated with steroids at least 1 year before LoA, while 68 (55.3%) had not; for 8 patients (6.5%) steroid treatment status before LoA was not known with certainty. When performing Kaplan-Meier analysis grouping by *SPP1* genotype and steroid treatment, no significant differences were observed by the log-rank test, although the observed effect of steroid treatment on median age at LoA tended to be greater in T/T patients (9.9 years in 44 untreated vs. 11.3 years in 29 treated) than in T/G-G/G patients (10.3 years in 33 untreated vs. 10.9 in 19 treated), showing a trend towards greater efficacy of steroid treatment in T/T patients, as previously suggested[25] (Fig 3D).

*LTBP4* SNPs were successfully genotyped in 137/145 patients in the LoA cohort, and LTBP4 haplotype could be phased with certainty in 135. The rs10880 T/T genotype and the IAAM/IAAM haplotype, which are in close LD, were associated to *earlier* median LoA: 9.9 years for T/T versus 10.9 years for C/C-C/T (n = 25 and 112 respectively, log-rank p = 0.058) (Fig 3B); and 9.7 years for IAAM/IAAM, 10.8 years for other haplotypes, and 11.1 years for VTTT/VTTT (n = 13, 83 and 39 respectively, log-rank test for IAAM/IAAM vs. all other haplotypes p = 0.037) (Fig 3C), in the opposite direction of association compared to Flanigan and colleagues’ findings[26]. Findings for the other individual *LTBP4* SNPs showed similar trends (data not shown). When grouping for concurrent effects of *LTBP4* genotypes and steroid treatment before LoA, a bigger difference in median LoA was observed within treated and untreated patients in the rs10880 C/C-C/T genotype group (9.9 years untreated vs. 11.9 years treated, n = 55 and 47 respectively) than in the TT genotype (9.9 years untreated vs. 10.9 years treated, n = 15 and 8 respectively) (Fig 3E), although the log-rank test for genotypes between treated patients was not significant. Due to the strong LD between rs10880 and the IAAM haplortype, the same median values of age at LoA were observed in the haplotype analysis (Fig 3F).

### Loss of ambulation and cardiomyopathy

**Fig 4:**
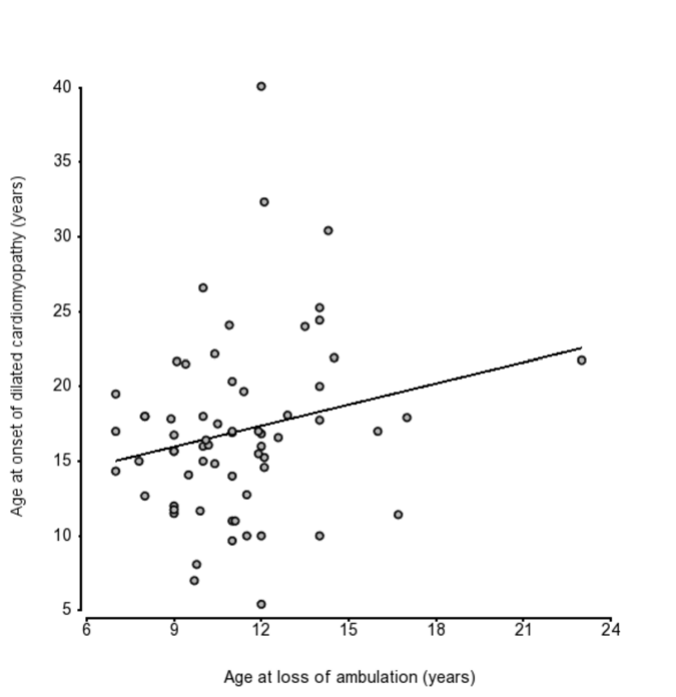
Scatter plot of age at LoA and DCM onset in 57 patients shows no strong correlation (r = 0.31).

In 57 patients for whom both DCM onset and LoA were observed during follow-up, we did not observe a protective effect on the heart of early loss of ambulation (r = 0.199, p = ns) (Fig 4).

## Discussion

Known genetic modifiers of skeletal muscle function in DMD were tested for association with DCM onset in a DMD cohort. As DCM is often asymptomatic and underdiagnosed in DMD[10], we adopted a stringent phenotype definition and standardized echocardiographic parameters, requiring a prior normal value in a narrow time window (1 year). Furthermore, phenotype definition integrates both reduced LV-EF, an expression of ventricular hypokinesis, and increased LV-VTD, denoting ventricular dilation even in the absence of ventricular function depression. While LV-EF strictly correlates with fractional shortening (FS), a widely used measure[6,8,10], including LV-VTD potentially increases diagnostic sensitivity.

A limit of this retrospective study is that only one of LV-EF or LV-EDV were available for some patients. However, average age at onset did not differ in these cases. Another limit is that age at DCM onset does not necessarily correlate with progression towards heart failure, which is variable and influenced by treatments. As treatment and follow-up were not standardized in this retrospective, multicentric study, capturing this variability in detail was not possible.

The natural history of thus-defined DCM in our population, depicted in the Kaplan-Meier plot (Fig 1A), places median onset at 20 years, 4~5 years later than a cohort similarly studied by Barber et al[8]. Mean age at onset in 71 patients developing DCM was also slightly later than in other studies: 16.5 years vs. 14.3 in Barber et al[8], and 15.4 in Jefferies et al[36] (also including Becker muscular dystrophy cases). The tendency to later onset cannot be ascribed to diagnostic delay, as we only included patients with normal echocardiography 1 year prior. Normal LV-EF cut-off at 50%, on the other hand, might be considered strict in comparison to other authors using 55%[8,10]. While we chose the higher specificity of a lower LV-EF threshold, this difference might delay onset.

DCM onset was distributed over a wide range of 5 to 40 years. Individual patients showed surprisingly early echocardiographic alterations, or, on the contrary, preservation of ventricular size and function well into adult age. This leads to hypothesize the presence of other precipitating or protective factors, including steroid treatment and genetic modifiers.

Our data do not confirm effectiveness of steroids in delaying DCM[6–8]; on the contrary, some “outlier” patients who never developed DCM, or developed it late in life, tended to be more frequently untreated (Fig 1B). This may reflect detrimental effects of steroids on the myocardium, promoting left ventricular fibrotic remodeling and dilation, as in prednisolone-treated *mdx* mice[12,13]. Our definition of DCM onset, including indicators of left ventricular dilation, might have been sensitive to volume overload and mineralocorticoid activity secondary to steroids, which might favor eccentric remodeling. Indeed, both ACEi[37] and aldosterone blockade[38] have been shown to improve DCM in DMD. Conversely, steroids might be effective in preventing progression to heart failure by other mechanisms.

When comparing DCM onset between *SPP1* genotypes, we observed a 5-year delay associated with the T/G-G/G genotype, although not statistically significant. As the G allele is expected to reduce *SPP1* expression[28], this trend would be in the direction suggested by over-expression experiments of *SPP1* in the murine myocardium, causing myocarditis and myocardial dilation[23]; but in the opposite direction compared to described effects of *SPP1* rs28357094 on skeletal muscle (greater weakness associated with the G allele)[19,24,25,29,39]. Perhaps relevant is that *SPP1* genotype may represent a pharmacogenetic locus, influencing response to steroids[25,29]. As steroids seem to have little effect on heart involvement in the population studied here, the effect of this pharmacogenetic locus may be obscured in the heart. In fact, the difference in median age at DCM onset was more marked in steroid treated patients (see Table 1), although still not statistically significant. Further investigations of underlying tissue-specific mechanisms would be warranted by stronger evidence of this genotype-phenotype association.

The rs10880 T/T genotype in the *LTBP4* gene, in LD with the IAAM haplotype, also showed a trend of association with delayed onset of cardiomyopathy, although not statistically significant (Fig 1B and 1C). If this trend were confirmed in independent populations, it could reflect reduced TGFβ signaling in homozygous T/T or IAAM patients, similar to what described by Flanigan and colleagues[26] in fibroblasts. This trend was clearer for rs10880 than other *LTBP4* SNPs, and apparently stronger for the full haplotype, although rarity of homozygous IAAM/IAAM individuals hindered analyses, suggesting a biological effect of the T1140M aminoacid change, within the TGFβ-binding domain of LTBP4.

In the literature about SNPs modifying LoA in DMD, both *SPP1* and *LTBP4* effects are suggested to depend on steroid therapy, as modifier of steroid response for *SPP1*[25] and as an additive effect for *LTBP4*[26]. Especially in the myocardium, where steroids have demonstrated a pro-fibrotic potential—at least in muscular dystrophy murine models[11–13]—a steroid-dependent effect of LTBP4 haplotype, modifying fibrosis through TGFβ signalling, represents an attractive hypothesis. In fact, when limiting DCM-free survival analyses to 73 steroid-treated patients, we observed a significant delay of onset in association to the rs10880 T/T genotype (Fig 2E). Chance of progression to heart failure would be a major factor in evaluating the risk/benefit balance of protracted steroid treatment, especially in the non-ambulatory phase, and our data suggest that *LTBP4* genotyping might be of aid to clinicians in stratifying risks. Further independent confirmations of this association are needed, before *LTBP4* genotyping is implemented in DMD clinical care with this purpose.

Survival analyses for LoA did not confirm expected associations in this cohort. *SPP1* rs28357094 genotypes showed no significant differences in median delay of LoA, although the differences of median values between steroid treated and untreated were higher in the T/T vs. T/G-G/G genotype (1.4 vs. 0.5 years), as previously suggested[25]. Surprisingly, the IAAM homozygote LTBP4 haplotype was associated with significantly *earlier* LoA—a finding in the opposite direction to that reported by Flanigan and colleagues, and replicated in 2 independent cohorts[25,27]. The cohort reported here is retrospective (average year of birth 1990), with relatively early median LoA for current standards (before 11 years of age), and a relatively low rate of steroid treatment while ambulatory (41.1%), due to the inclusion of several patients followed in a time when steroid therapy was not a universal standard of care. Earlier LoA might reduce statistical power for validation, as a more severe phenotype compresses the relatively small differences due to common polymorphisms. Furthermore, if genetic modifiers, as suggested, influence treatment response rather than disease progression directly, effects might be reduced in a population with a low treatment rate.

Lastly, we did not observe significant correlations between LoA and DCM onset, as suggested by authors postulating a protective effect of limited exertion[3,4], supporting a model of independent skeletal muscle and myocardic dystropathology in DMD. This observation highlights the fact that while both descriptive clinical studies and interventional clinical trials have so far concentrated on ambulation and ambulatory endpoints, loss of ambulation may not be a good predictor of long-term prognosis and survival. Also, different genetic modifiers could be acting with tissue-specific mechanisms that differentially influence sub-phenotypes (e.g. muscular weakness, cardiomyopathy) in a diverse time-frame within the same disease.

The potential impact of our findings, if validated in independent cohorts, should be interpreted in the context of genotype-phenotype studies, which have refined the correlation between different truncating *DMD* mutations and DMD natural history[40,41], and genetic modifier studies cited above. As the field of rare genetic diseases shifts to a personalized medicine approach, the precise definition of the disease-causing mutation, together with targeted genotypization at established modifier loci, could help provide prognostic indications to patients and families, and fine-tune standards of care to individual patient characteristics. For instance, we identified a putative predictive value of the *LTBP4* rs10880 genotype for delay of DCM onset with steroid treatment, which could have a role in deciding if and how long to maintain treatment in non-ambulatory patients.

An even more pressing issue is the stratification of participants in clinical trials for new molecular and genetic treatments. Common variants in genes involved in inflammation and remodeling pathways, as those studied here, could be probably relevant for disease progression and efficacy of dystrophin-restoring agents. Subsequently, a sensible approach would be to ensure by genotypization that allele frequencies for relevant loci are not too different from the general population in both treated and placebo cohorts.

In conclusion, we observed trends towards a protective effect of the dominant G allele at *SPP1* rs28357094 and recessive T allele at *LTBP4* rs10880, which was statistically significant in steroid-treated patients for *LTBP4* rs10880. On the other hand, an independent effect of steroid treatment in delaying DCM onset (defined as the age at the first finding of LV-EF <50% or LV-EDV > 70 mL/m^2^) was not confirmed in this population.

## Supporting Information

S1 Fig Scatter plot of left ventricular ejection fraction (EF) values by age (years) and rs28357094/rs10880 genotypes. The dashed line marks the cut-off for normal EF (>50%).
(PDF)

S2 Fig Scatter plot of left ventricular end diastolic volume (EDV) values by age (years) and rs28357094/rs10880 genotypes. The dashed line marks the cut-off for normal EDV (<70 mL/m^2^)
(PDF)
