# Supplementary material for: Genetic Modifiers of Duchenne Muscular Dystrophy and Dilated Cardiomyopathy
Source: PLoS One. 2015 Oct 29;10(10):e0141240. doi: 10.1371/journal.pone.0141240 (PMC4626372; doi:10.1371/journal.pone.0141240)
Supplement: S2 Fig — The dashed line marks the cut-off for normal EDV (<70 mL/m2) (PDF) [file pone.0141240.s002.pdf]

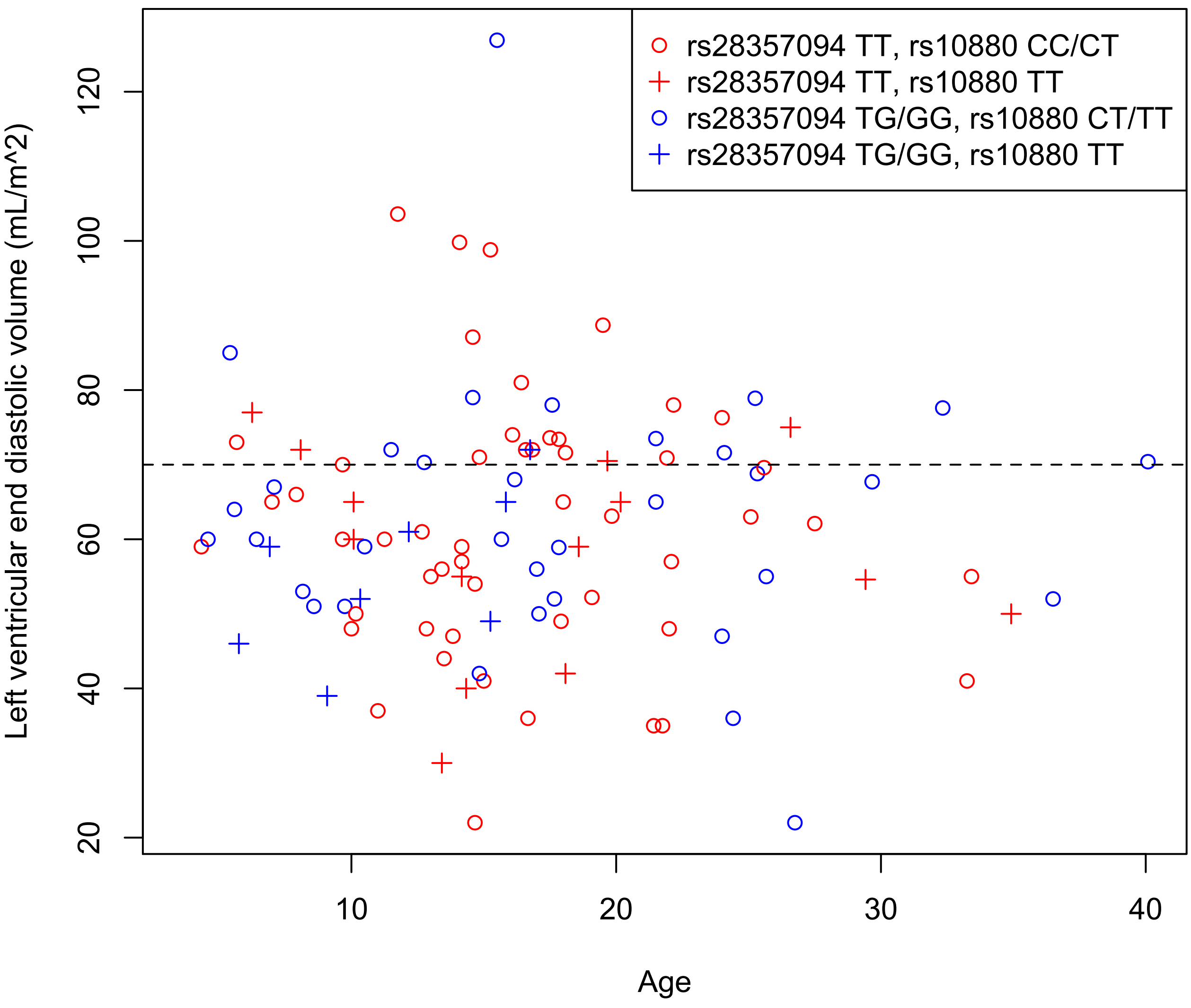

rs28357094 TT, rs10880 CC/CT
rs28357094 TT, rs10880 TT
rs28357094 TG/GG, rs10880 CT/TT
rs28357094 TG/GG, rs10880 TT
Left ventricular end diastolic volume (mL/m^2)
Age
20
40
60
80
100
120
10
20
30
40

Supplemental Figure 2. Scatter plot of left ventricular end diastolic volume (EDV) values by age (years) and rs28357094/rs10880 genotypes. The dashed line marks the cut-off for normal EDV (<70 mL/m^2).
